# Supplementary material for: Metastatic Tumor Cell-Specific FABP7 Promotes NSCLC Metastasis via Inhibiting β-Catenin Degradation
Source: Cells. 2022 Feb 25;11(5):805. doi: 10.3390/cells11050805 (PMC8909100; doi:10.3390/cells11050805)
Supplement: Supplementary file 1 [file cells-11-00805-s001.zip › cells-1593659-supplementary/Supplementary Figure legends.pdf]

Supplementary Figure legends

Figure S1. Quality control and main cell types identification of single-cell RNA sequencing data.

(**A**) Preliminary quality control. Thresholds of detected genes per cell (nFeature_RNA), detected counts per cell (nCount_RNA), and percentage of mitochondrial genes counts (Percent.mt) are shown. (**B**) and (**C**) Principal component analysis based on different cell cycles (**B**) or mitochondrial genes expression (**C**). Distinctions of Cells distribution reflect the influence of cell cycling (**B**) or mitochondrial genes expression (**C**) on clustering. (**D**) UMAP visualization of unsupervised primary clustering. Clusters are indexed in order of cells amount. (**E**) and (**F**) Identifications of primary clustering. Cell-defining markers expressions of each cluster (**E**) were used to identify well-known cell types. Cell-defining markers expressions of identified cell types (**F**) were used to examine the result of identification.

Figure S2. Identification and cell proportion summary of cell subtypes.

(**A-C**) Cell subtypes identification. Subtypes of NK and T cells (**A**), myeloid cells (**B**), and plasma cells (**C**) were independently clustered and identified. Cell-defining markers expressions of indicated cell types were used to examine the results of identification. (**D**) Cell proportion summary of all cell subtypes in the tumor tissue.

Figure S3. Expression patterns and first progression analyses of FABP3-7.

(**A**) FABP3-7 expressions of all cells shown by heatmap. Expression levels are labeled by the intensity of red color. (**B**) Average expressions of FABP3-7 in all main cell types split by adjacent (left) or tumor tissues (right). (**C**) Differentially expressed FABPs in metastatic vs. non-metastatic tumor tissues. Expression changes of FABP3 and FABP6 are not significant and thus not presented. The adjusted p-value of FABP7 in tumor cells is approximate to 0, so it has been assigned to 10e-200 only for visualization purpose. (**D**) Kaplan-Meier first progression (FP) analyses of FABP3-7 in NSCLC based on KM-plotter.

Figure S4. Metastasis-related GO analysis and GSEA.

(**A**) GSEA enrichments of scRNAseq for metastasis-related gene sets. (**B**) and (**C**) GO analysis (**B**) and GSEA (**C**) of TCGA NSCLC bulk-seq for metastasis-related gene sets.

Figure S5. Detection of FABP7 on cell proliferation.

Representative images of three independent reproducible experiments and quantitation of cells colony formation by FABP7 over-expression in A549 and H1975.

Figure S6. GO/KEGG analyses and qPCR for lipid-related pathways.

(**A**) GO and KEGG analyses for lipid-related pathways (based on DEGs of metastatic vs. non-metastatic all cell types). (**B**) GO and KEGG analyses for lipid-related pathways (based on DEGs of FABP7-positive vs. FABP7-negative tumor cells). (**C**)

The qPCR validations of lipid droplets markers.

Figure S7. GO analysis and GSEA for Wnt signaling pathway.

(**A**) and (**B**) GO analysis (**A**) and GSEA (**B**) of TCGA NSCLC bulk-seq for the Wnt signaling pathway.
