# Supplementary figures and images for: Metastatic Tumor Cell-Specific FABP7 Promotes NSCLC Metastasis via Inhibiting β-Catenin Degradation
Source: Cells. 2022 Feb 25;11(5):805. doi: 10.3390/cells11050805 (PMC8909100; doi:10.3390/cells11050805)

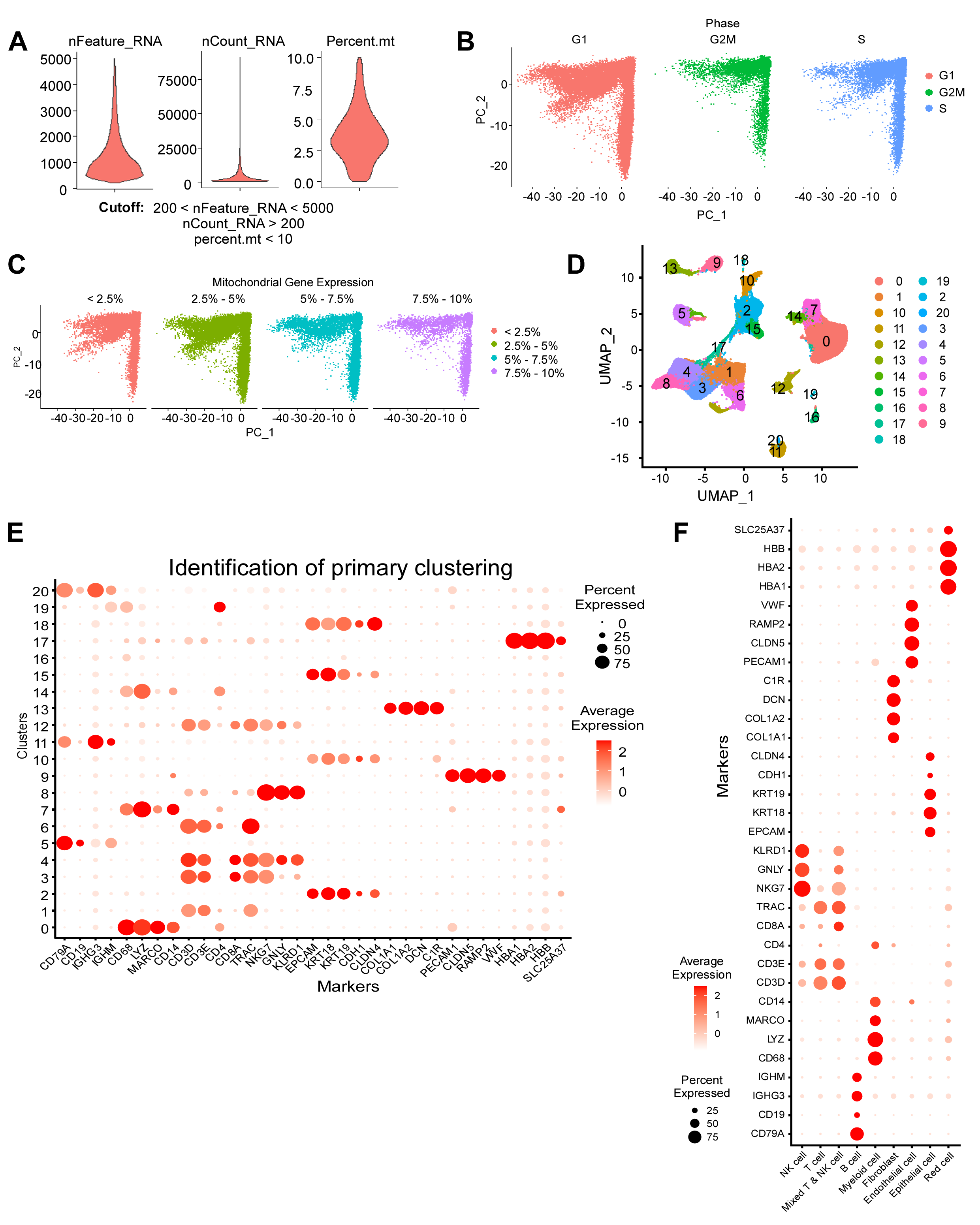

Supplement: Supplementary file 1 [file cells-11-00805-s001.zip › cells-1593659-supplementary/FigureS1.tif]

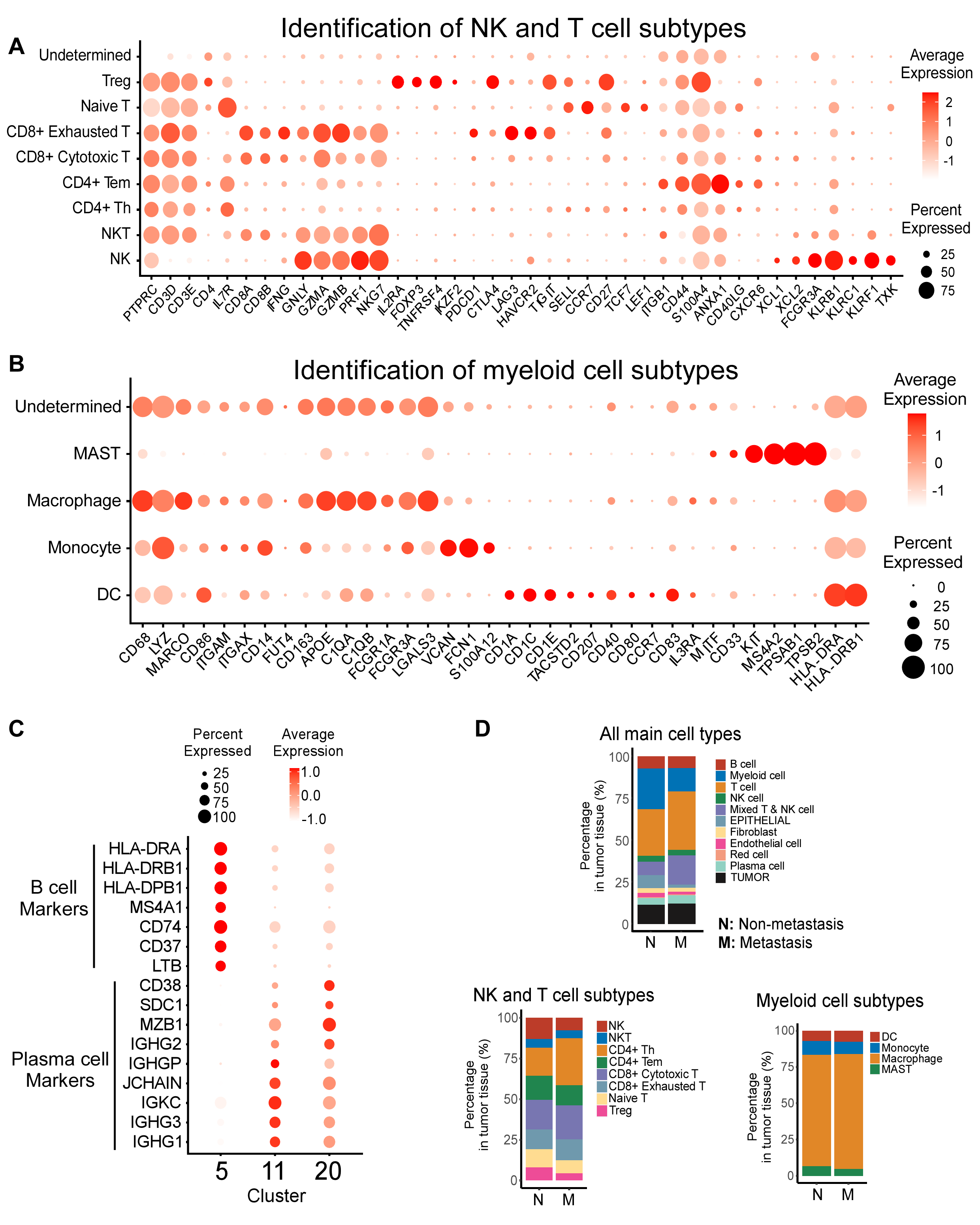

Supplement: Supplementary file 1 [file cells-11-00805-s001.zip › cells-1593659-supplementary/FigureS2.tif]

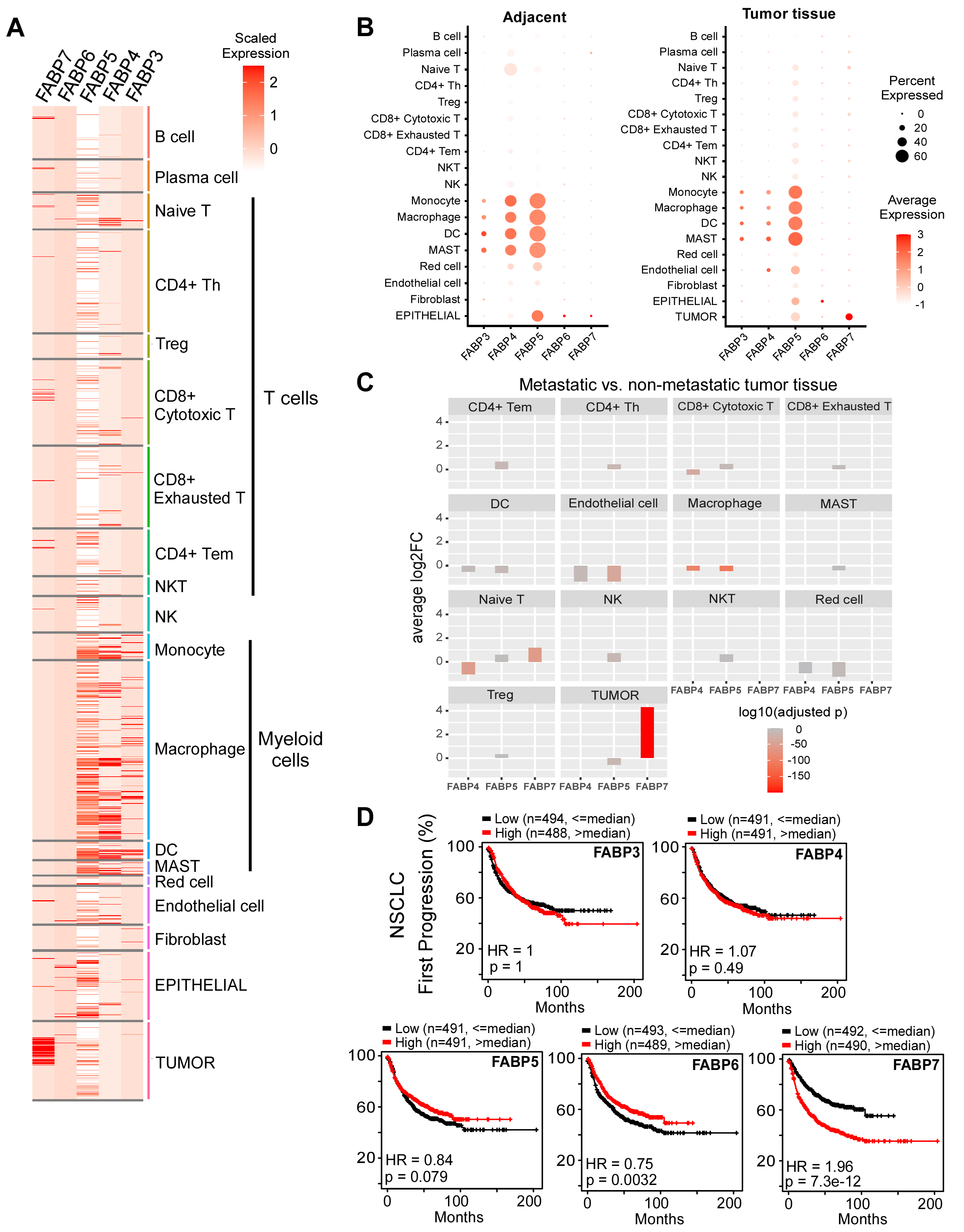

Supplement: Supplementary file 1 [file cells-11-00805-s001.zip › cells-1593659-supplementary/FigureS3.tif]

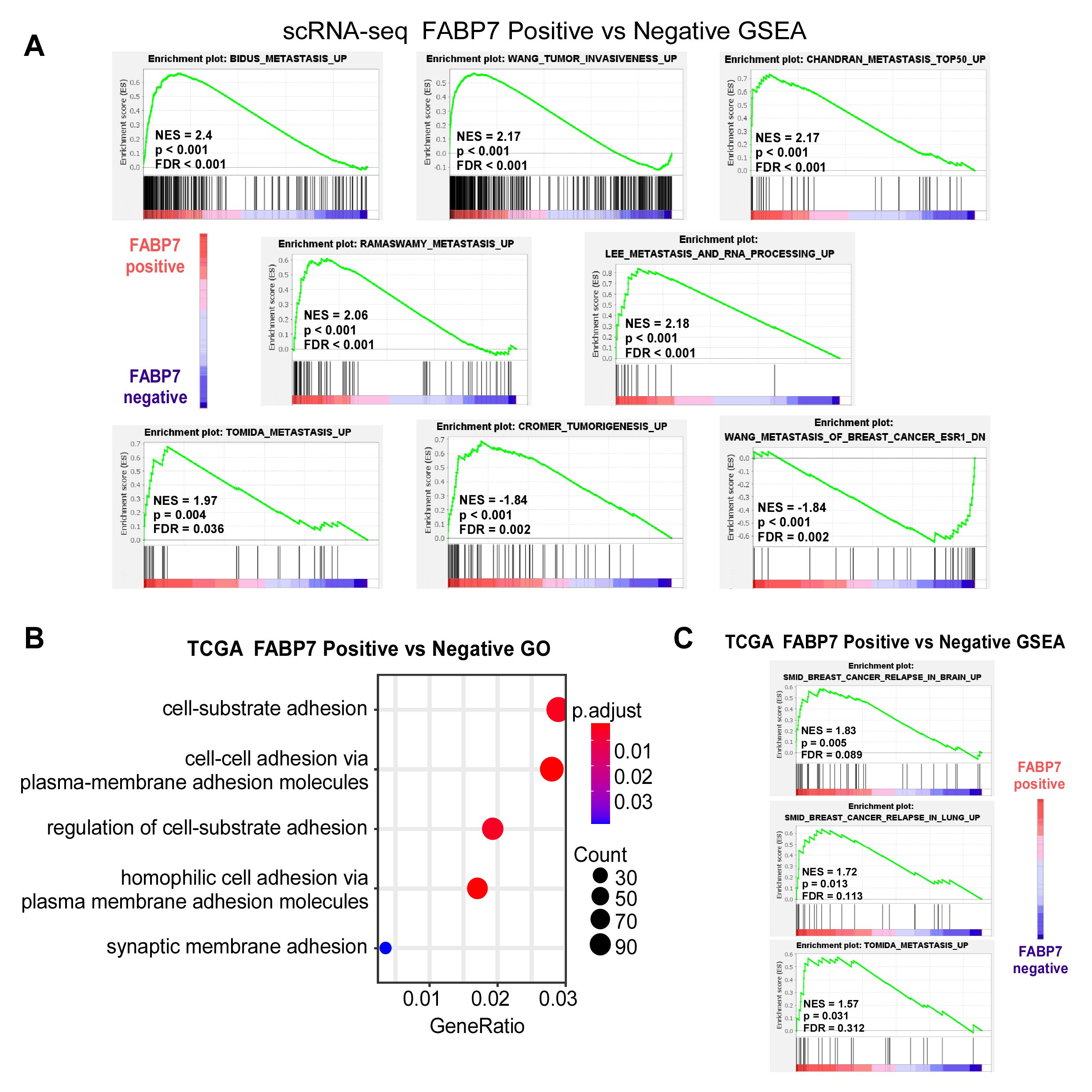

Supplement: Supplementary file 1 [file cells-11-00805-s001.zip › cells-1593659-supplementary/FigureS4.tif]

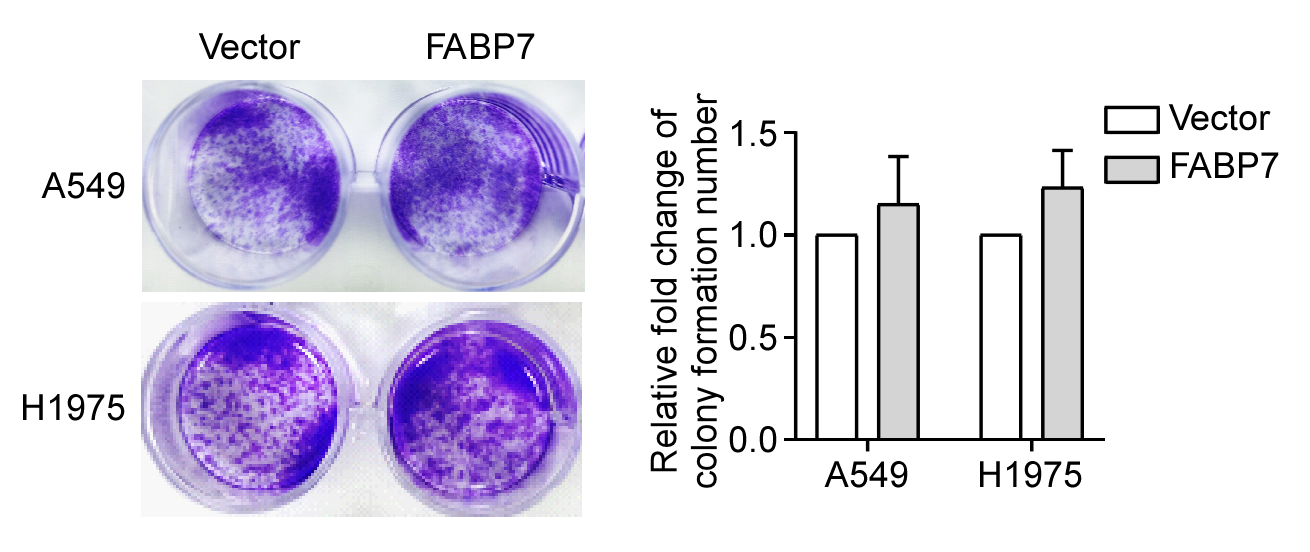

Supplement: Supplementary file 1 [file cells-11-00805-s001.zip › cells-1593659-supplementary/FigureS5.tif]

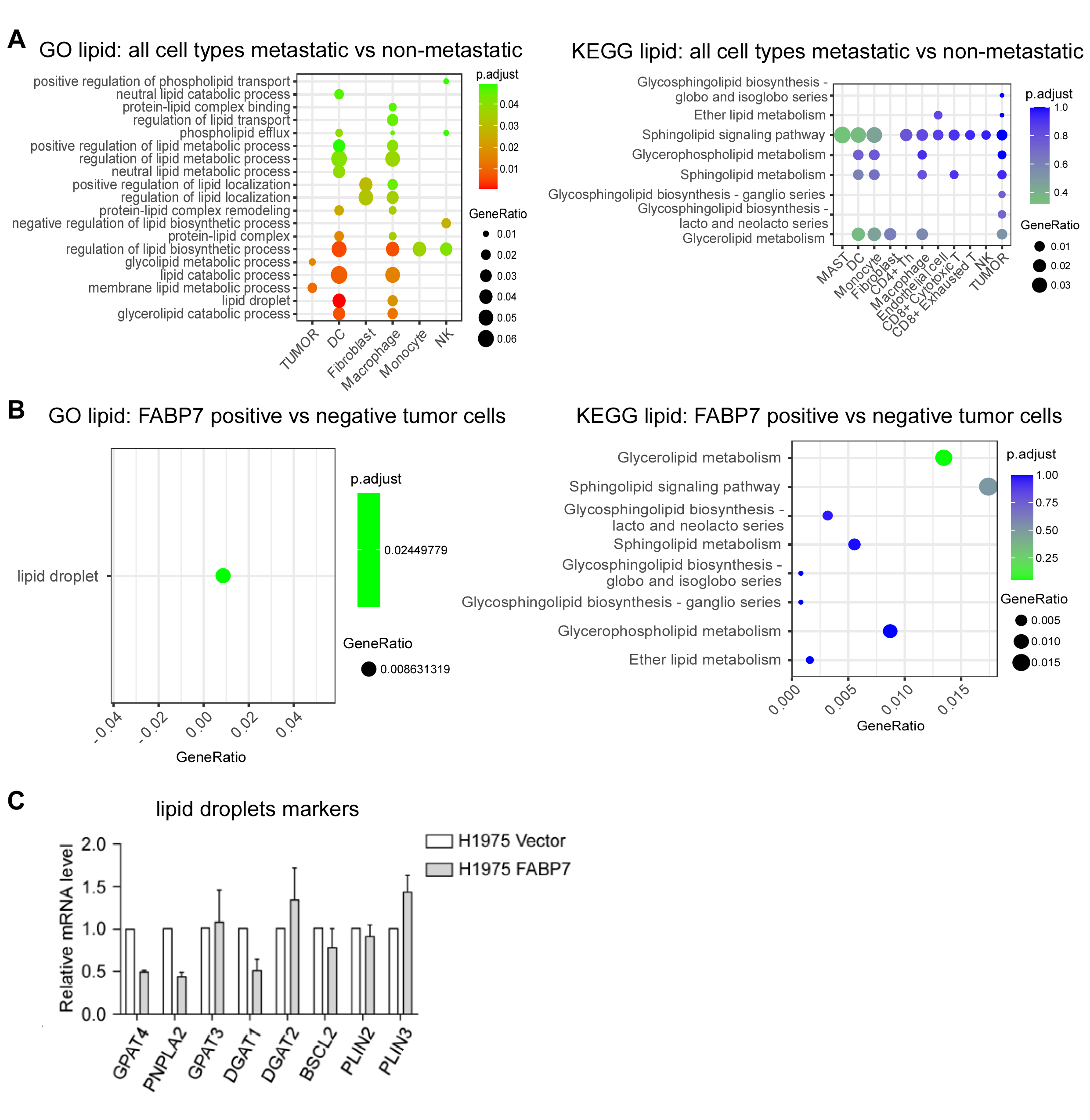

Supplement: Supplementary file 1 [file cells-11-00805-s001.zip › cells-1593659-supplementary/FigureS6.tif]

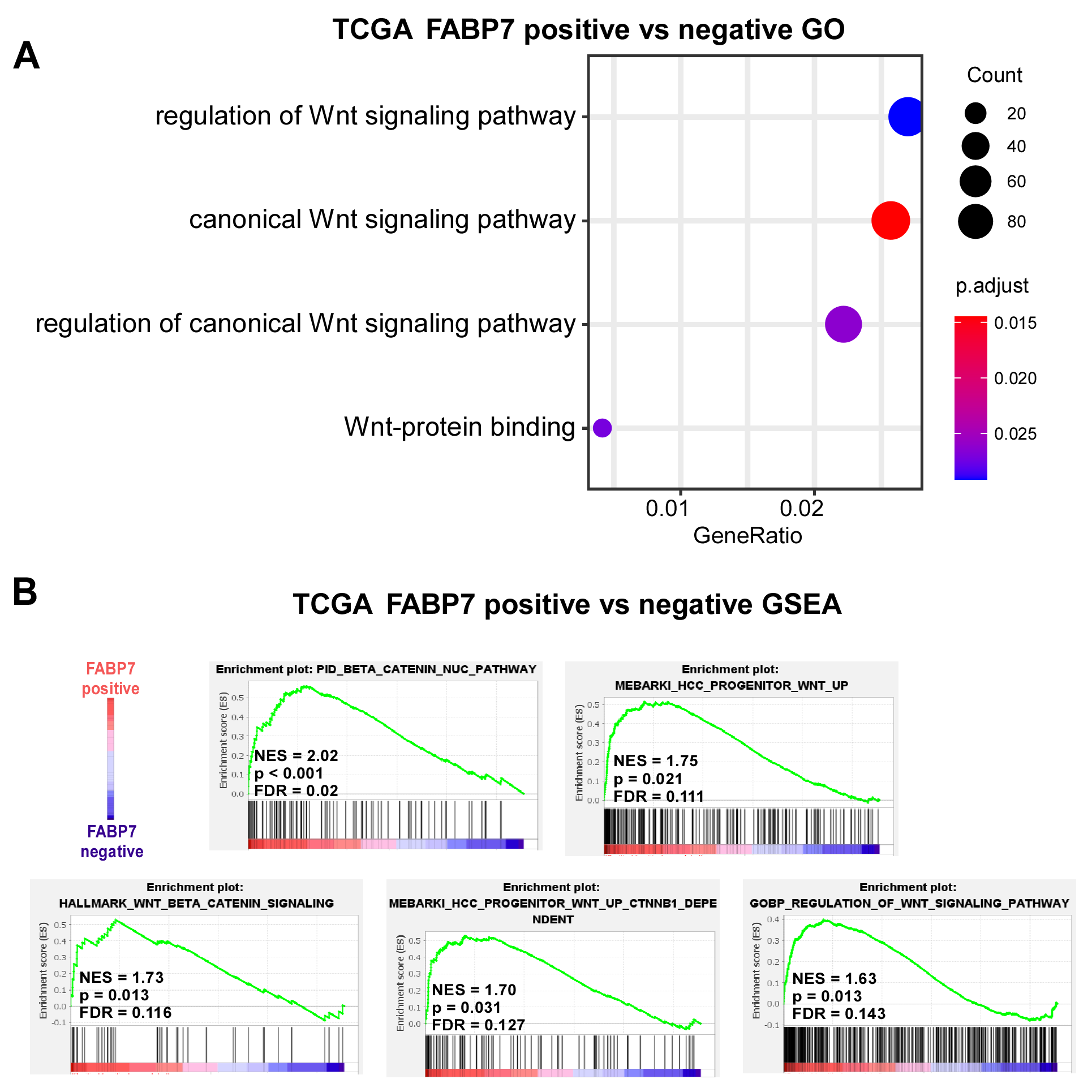

Supplement: Supplementary file 1 [file cells-11-00805-s001.zip › cells-1593659-supplementary/FigureS7.tif]
